# Comparative optimization of combinatorial CRISPR screens

**DOI:** 10.1038/s41467-022-30196-9

**Published:** 2022-05-05

**Authors:** Ruitong Li, Olaf Klingbeil, Davide Monducci, Michael J. Young, Diego J. Rodriguez, Zaid Bayyat, Joshua M. Dempster, Devishi Kesar, Xiaoping Yang, Mahdi Zamanighomi, Christopher R. Vakoc, Takahiro Ito, William R. Sellers

**Affiliations:** 1 Broad Institute of Harvard and MIT, Cambridge, MA USA; 2 Cold Spring Harbor Laboratory, Cold Spring Harbor, NY USA; 3 Harvard Medical School, Boston, MA USA; 4 Department of Medical Oncology, Dana-Farber Cancer Institute, Boston, MA USA; 5 Scorpion Therapeutics, Boston, MA USA

**Keywords:** CRISPR-Cas9 genome editing, Genomics

## Abstract

Combinatorial CRISPR technologies have emerged as a transformative approach to systematically probe genetic interactions and dependencies of redundant gene pairs. However, the performance of different functional genomic tools for multiplexing sgRNAs vary widely. Here, we generate and benchmark ten distinct pooled combinatorial CRISPR libraries targeting paralog pairs to optimize digenic knockout screens. Libraries composed of dual *Streptococcus pyogenes* Cas9 (spCas9), orthogonal spCas9 and *Staphylococcus aureus* (saCas9), and enhanced Cas12a from *Acidaminococcus* were evaluated. We demonstrate a combination of alternative tracrRNA sequences from spCas9 consistently show superior effect size and positional balance between the sgRNAs as a robust combinatorial approach to profile genetic interactions of multiple genes.

## Abstract (web summary)

Combinatorial CRISPR screens can be utilized to identify genetic interactions and functional redundancies of multiple genes. Here, the authors benchmark ten digenic CRISPR technologies and identify novel Cas9 tracrRNA combinations that show superior performance.

## Introduction

Comprehensive maps of cancer dependencies are being elaborated by genome-wide CRISPR and RNAi screens across over 900 cell lines^1–3^. The next potential wave of functional genomic discoveries and identification of new therapeutic approaches for cancer will likely emerge through the identification of combinatorial genetic interactions including context-specific dependencies of redundant paralogous genes missed in single-gene knockout studies. Both approaches require robust technologies to profile simultaneous knockouts of two or more genes.

Recent advances in multiplexed combinatorial CRISPR screens have allowed for interrogation of digenic or trigenic knockout dependencies in human cell lines. Initial studies utilized vectors to express two sgRNAs in *Streptococcus pyogenes* Cas9 (spCas9)-expressing cell lines^4–8^. Subsequently, utilization of spCas9 and *Streptococcus aureus* (saCas9) improved the library quality by overcoming the challenge of recombination as orthologous Cas9 systems have distinct tracrRNA sequences^9,10^. Recently, the emergence of enhanced or optimized Cas12a (enCas12a) systems has also shown promise in combinatorial genetic screens^11–15^.

Despite these advances, understanding which combinatorial CRISPR technologies lead to efficient and robust digenic knockouts remains a challenge. Meta-analyses of previous studies have been difficult as the CRISPR libraries between these studies are composed of different gene sets, sgRNA designs and screened in different cell lines^16^. In this study, we evaluated ten distinct digenic libraries targeting single genes and overlapping paralogous pairs using three major CRISPR systems (spCas9-saCas9, enCas12a, and combinations of alternative spCas9 tracrRNA sequences) to try and determine an optimal combinatorial CRISPR system. We identified a combination of unique tracrRNA sequences that outperforms previously published systems.

## Results

### CRISPR systems for dual-knockout screens

**Fig. 1:**
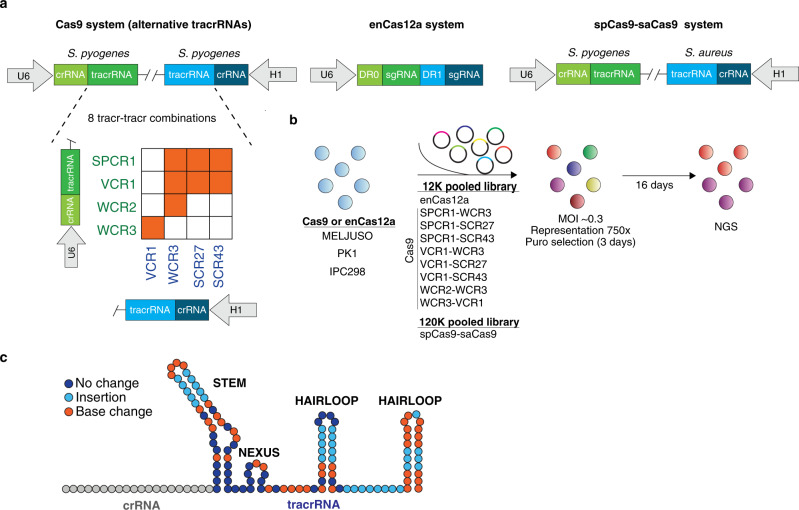
Comparison of combinatorial CRISPR libraries. **a** Three main combinatorial CRISPR systems evaluated: eight distinct combinations of alternative spCas9 tracrRNAs sequences (orange boxes represent libraries generated), enCas12a, and orthologous spCas9-saCas9 system. **b** Schematic representation of the CRISPR screen pipeline. Ten distinct libraries are screened in IPC298. spCas9*-*saCas9, enCas12a, VCR1-WCR3, and WCR2-WCR3 libraries are screened in PK1 and MELJUSO. **c** Schematic of sgRNA constant regions with indicated alterations compared to original spCas9 tracrRNA. Dark blue represents canonical sequence; light blue represents regions where insertions are introduced; orange represents base changes.

To investigate how different CRISPR systems influence the overall quality of dual-knockout screens, we developed nine pooled dual CRISPR libraries targeting 616 genes (6 sgRNAs per gene) and 454 paralogous pairs (18 sgRNA combinations for each paralog pair) (Supplementary Fig. 1a). We compared the performances of three combinatorial CRISPR systems: enCas12a (also referred to as *Cpf1*), dual spCas9 with various combinations of alternative tracrRNA sequences, and orthologous spCas9 and saCas9 systems using published datasets (Fig. 1a, b)^10^. The libraries included an overlapping panel of nonessential and pan-essential genes and paralog pairs to enable the assessment of single-gene and digenic knockouts efficacy (Supplementary Data 1–4).

A major advantage of utilizing a spCas9 system is the availability of genome-wide CRISPR screens from DepMap to select validated sgRNAs^17^. To this end, we examined the combinations of new and published alternative tracrRNA sequences with various mutations and insertions to overcome the recombination challenges of using the same or similar tracrRNA sequences (see “Methods” for rationale; Fig. 1c and Supplementary Fig. 1b, c)^18,19^. For the spCas9 sgRNA design, we prioritized sgRNAs from the Avana library that exhibited high agreement across 770 cell lines followed by sgRNAs targeting functional domains (PFAM) from Rule Set2 (Supplementary Fig. 1d–f)^20–22^. For the enCas12a library, we selected sgRNAs designed by enPAM+GB (https://broad.io/crispick) and prioritized sgRNAs targeting PFAM domains (Supplementary Fig. 1g, h)^12^. After cloning, libraries using variable tracrRNA sequences for Cas9 or direct repeat (DR) sequences for enCas12a exhibited optimal library distribution (Supplementary Fig. 1i) and a low recombination rate that was undetectable by gel electrophoresis (Supplementary Fig. 1j).

### Optimized alternative Cas9 tracrRNA combinations exhibit robust single-gene knockout

**Fig. 2:**
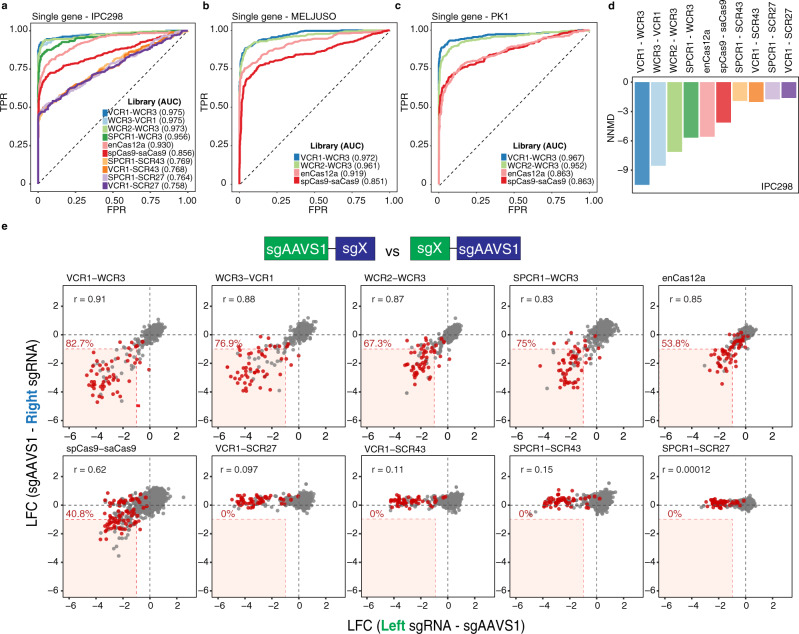
Performance of combinatorial systems at the single-gene knockout level. **a**–**c** The single-gene receiver operating characteristic (ROC)—area under the curve (AUC) curves derived from pan-essential and nonessential genes across distinct libraries in (**a**) IPC298, (**b**) MELJUSO, and (**c**) PK1. AUC values are indicated in parentheses. **d** Separation between gene scores for common essential genes and nonessential genes computed using null-normalized median difference (NNMD). **e** Correlation between the average LFC of right versus left sgRNAs coupled with sgAAVS1 cutting control in IPC298. Pearson’s correlation coefficient is indicated (*r* value) in black. Percentage of pan-essential genes with LFC less than −1 for sgRNAs in both right and left positions are indicated in red. Pan-essential genes are annotated by red dots. Source data are provided in the Source Data file.

To assess the efficacy of the ten distinct combinatorial library systems, we first screened the libraries in an *NRAS*-mutant cutaneous melanoma cell line, IPC298. All the screens exhibited a high correlation among the three biological replicates (Supplementary Fig. 2a). The performance of the libraries was first evaluated at the single-gene level by receiver operating characteristic (ROC)—area under the curve (AUC) and null-normalized mean difference (NNMD) analyses based on six sgRNAs paired with AAVS1 sgRNA for a predefined set of core essential and nonessential genes allowing us to determine the true and false-positive rates. We observed that several alternative spCas9 systems (VCR1-WCR3, WCR3-VCR1, WCR2-WCR3, and SPCR1-WCR3) outperformed enCas12a and orthologous Cas9 systems (Fig. 2a, d). The tracrRNA combinations utilizing the SCR27 or SCR43 tracrRNAs exhibited the lowest performance (discussed below). Based on these results, the two unique tracRNA pairs that performed the best were compared to the enCas12a and orthologous Cas9 system in two additional cell lines, MELJUSO and PK1, where we observed similar results (Fig. 2b, c). In addition, a comparison to the genome-wide Avana screens showed that VCR1-WCR3 and WCR3-VCR1 were the only libraries that exhibited stronger depletion of pan-essential genes than observed in the Avana library (Supplementary Fig. 2b).

### Evaluation of sgRNA positional effect

A major challenge in combinatorial CRISPR screens is maintaining efficient and balanced knockouts between the two sgRNAs. We observed a high correlation between the left and right sgRNA except for libraries utilizing the SCR27 and SCR43 tracrRNAs (Fig. 2e). This was in concordance with their poor performance in the separation of essential and nonessential genes. These data suggest that the SCR27 and SCR43 tracrRNAs are inert potentially resulting from the introduction of several missense mutations from the originally published sequences to enable our cloning strategies. The VCR1-WCR3 library exhibited the highest percentage of pan-essential genes with LFC less than −1 by both sgRNAs (82.7%) and the highest correlation coefficient (*r* = 0.91) suggesting that this tracrRNA combination is the most balanced and efficacious.

**Fig. 3:**
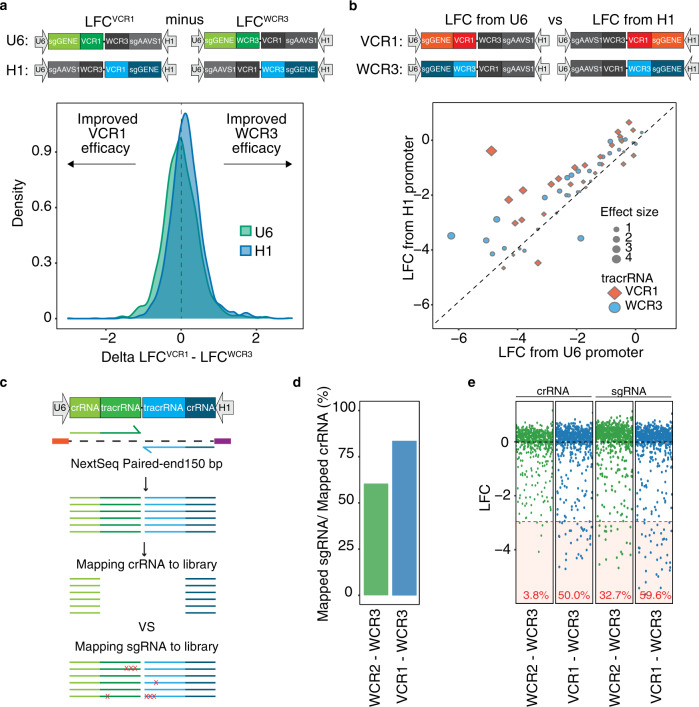
Positional effects and recombination rate on gene targeting. **a** Evaluation of tracrRNA effect calculated by the difference in LFC between sgRNAs utilizing the VCR1 and WCR3 tracrRNAs from the VCR1-WCR3 and WCR3-VCR1 screens in IPC298. Analyses separated by promoters expressing the sgRNA. **b** Evaluation of the promoter effect by correlation between the LFC from the U6 and H1 promoter. For ten genes, same crRNA sequences are used on both left and right position to remove crRNA variability. Analyses separated by tracrRNA used. **c** Schematic illustration evaluating the recombination rate by next-generation sequencing (NGS) reads. crRNA and tracrRNA in both positions were sequenced by NextSeq paired-end 150 bp. Reads were either mapped to crRNA only or crRNA+tracrRNA (sgRNA). **d** Recombination rate calculated by percent of reads mapped between sgRNA to crRNA in pDNA for WCR2-WCR3 (green) and VCR1-WCR3 (blue). **e** LFC calculated by reads mapped to crRNA or sgRNA. The percentage of pan-essential genes with LFC less than −3 are indicated in red. Source data are provided in the Source Data File.

As the VCR1-WCR3 library exhibited the most robust single-gene knockout efficacy, we utilized this tracrRNA combination to look for subtle differences between the two tracrRNAs. To this end, we generated libraries where the two tracrRNAs were flipped (VCR1-WCR3 compared to WCR3-VCR1) while maintaining the same crRNA sequences and promoters. There was no detectable alternation in efficacy between the VCR1 and WCR3 tracrRNAs as the LFC difference between VCR1 and WCR3 resulted in normal distribution centered around zero (Fig. 3a). We next examined the effects of the U6 and H1 promoters driving the expression of the two sgRNAs. To enable a direct comparison of the promoters while maintaining the same crRNA sequence coupled to the tracrRNAs, we included the same crRNA sequences for 10 genes under both the U6 and H1 promoters. Although the magnitude of LFC between the two promoters was similar, we consistently detected stronger depletion in sgRNAs driven under the U6 promoter using both the VCR1 or WCR3 tracrRNA as previously described (Fig. 3b)^23^. These data together suggest that the positional effects between the two sgRNAs are minimal, and that the slight observed bias is likely driven by promoter differences.

### Homology of tracrRNA sequence contributes to high recombination rate

Notably, we observed stronger depletion by the sgRNAs utilizing the WCR3 tracrRNAs in the VCR1-WCR3 library compared to the same sgRNAs using the WCR3 tracrRNA in the WCR2-WCR3 library despite having the same promoter, crRNA sequence, and tracrRNA composition (Fig. 2e). We hypothesized that the decrease in WCR3 performance for the WCR2-WCR3 library might be potentially due to an increased recombination rate between these two more homologous tracrRNA sequences. To evaluate the rate of recombination more systematically, we extended the sequencing reads through the tracrRNAs for VCR1-WCR3 and WCR2-WCR3 pDNA and gDNA (Fig. 3c). The ratio of reads mapped to the crRNA and tracrRNA (sgRNA) to reads mapped only to the crRNA represents the potential rate of homologous recombination at the tracrRNA. In this analysis, we observed a higher rate of recombination for the WCR2-WCR3 library compared to the VCR1-WCR3 library (Fig. 3d). In addition, when we calculate the LFC for reads mapped to the sgRNA instead of only just the crRNA, the performance of the WCR2-WCR3 library significantly improved (Fig. 3e). These data together suggest that a higher recombination rate between the WCR2-WCR3 tracrRNA contributes to the decreased knockout performance compared to VCR1-WCR3 when measuring LFC using the crRNA. Given the cost of longer read sequencing, the reduction in recombination rate is a significant factor for library design and use.

### sgRNA selection improves enCas12a and Cas9 performance

The *Acidaminococcus* Cas12a variants (enCas12a) exhibit increased activity and expansion of protospacer-adjacent motif (PAM) preference^12–15^. As previously described^12,14^, in our screen sgRNAs utilizing the canonical PAM sequence (TTTV) showed increased enCas12a activity indicated by depletion of pan-essential genes (Supplementary Fig. 3a). In addition, while we selected enCas12a sgRNAs based on the weighted sum of on-target and off-target ranks for our library^12^, the performance of sgRNAs based on only the high on-target rank is also improved (Supplementary Fig. 3b). Together, these data suggest that improvement in sgRNA selection could enhance the performance of the enCas12a system.

For spCas9 sgRNA design, the highest prioritization was given to “pre-validated” sgRNAs from the Avana library. To this end, initial analyses confirmed our hypothesis that these sgRNAs are associated with superior performance compared to Rules Set2 sgRNAs indicated by depletion of pan-essential genes (Supplementary Fig. 3c). These latter data argue for continued curation of “robustly” validate sgRNAs as a future means to improve library performance and reduce library sizes.

### Comparison of double knockout efficiency

**Fig. 4:**
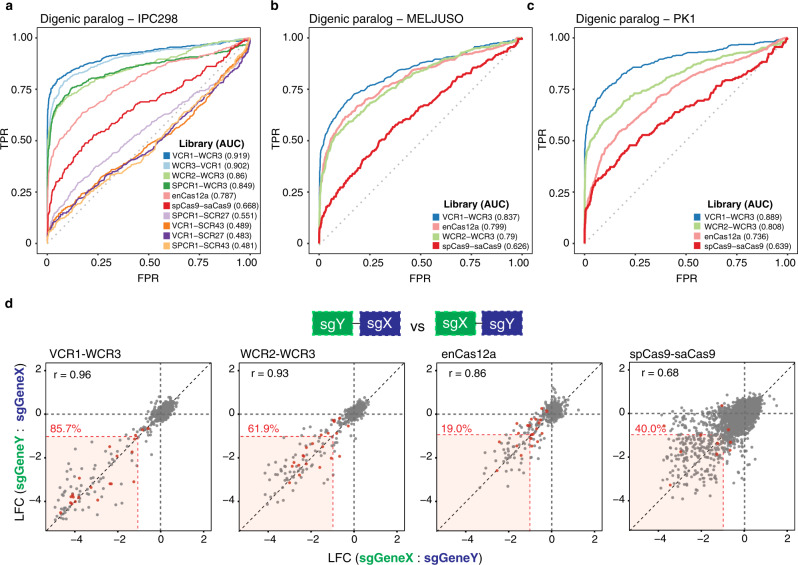
Benchmarking of dual knockouts by pan-essential paralog pair. **a**–**c** The ROC-AUC curves derived from pan-essential and nonessential paralog pairs across distinct libraries in (**a**) IPC298, (**b**) MELJUSO, and (**c**) PK1. AUC values are indicated in parentheses. **d** Correlation between the average LFC of dual knockouts with inverted positions of sgRNAs in IPC298. Pearson’s correlation coefficient is indicated (*r* value) in black. Percent of pan-essential paralog pairs with LFC less than −1 for both sgRNAs in positions are indicated in red. Pan-essential paralog pairs are annotated by red dots. Source data are provided in the Source Data File.

**Fig. 5:**
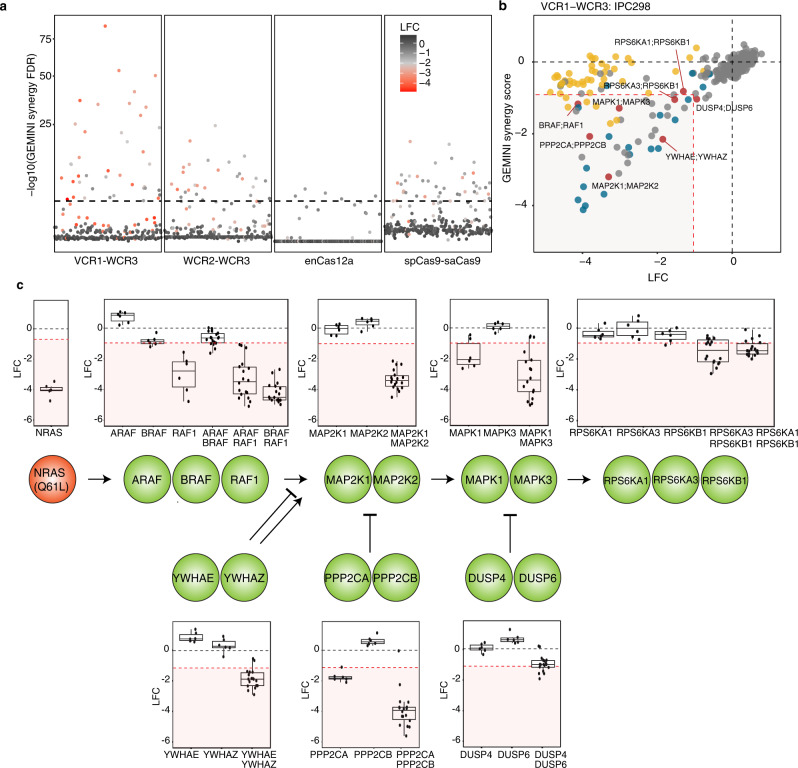
Positive and negative regulators of the MAPK pathway exhibit synergistic dependencies in *NRAS*-mutant melanoma. **a** Manhattan plot of FDRs corresponding to GEMINI synergy scores and color-coded by the LFC of dependencies for paralog pair knockouts in IPC298. The dotted line represents the FDR of 1e-3. **b** Scatter plot of GEMINI synergy score and LFC of dual knockout in IPC298. Curated paralog pairs of the MAPK pathway are annotated by red dots. Pan-essential paralog pairs or genes are annotated by blue or yellow dots, respectively. **c** LFC of single or combinatorial gene knockouts of paralog pairs associated with MAPK signaling pathway in IPC298 (*n* = 3 biological replicates; 6 independent sgRNAs per gene and 18 independent sgRNA combinations per gene pair). The centerline, lower hinge, and upper hinge correspond to the 50th, 25th, and 75th percentiles, respectively. The upper and lower whiskers extend from the upper and lower hinges to the largest and smallest values no further than 1.5 * IQR (interquartile range). Source data are provided in the Source Data File.

To evaluate the efficacy of the different library systems in the generation of the double knockouts, we defined essential paralog pairs from the genome-wide single-gene knockout studies where lethal interactions arise from the genetic inactivation, through mutation, deletion, or transcriptional downregulation of one member of a pair of paralogous genes leading to dependence on the remaining paralog (Supplementary Data 2)^10^. Nonessential pairs were defined as non-expressed paralog pairs across Cancer Cell Line Encyclopedia (CCLE). In general, the rank order of the ROC-AUC analyses of the dual paralog knockouts mirrored the single-gene knockout with a panel of alternative dual spCas9 outperforming the enCas12a, spCas9-saCas9, and any library utilizing the SCR27 or SCR43 tracrRNAs (Fig. 4a–c). In addition, panels of the dual spCas9 system and enCas12a system exhibited a high correlation between the two sgRNAs compared to the spCas9-saCas9 system which exhibited bimodal efficiency (Fig. 4d). However, VCR1-WCR3 consistently outperforms the rest of the libraries based on the magnitude and percent of pan-essential pairs exhibiting robust depletion (Fig. 4d and Supplementary Fig. 4a) as well as the number of synergistic dependencies observed (Fig. 5a). The difference in library performance across cell lines are predominantly attributed to the intrinsic Cas9 or Cas12 activities for individual cell lines (Supplementary Fig. 4b) though other cell line-specific factor likely underlie cell line-to-cell line variation. The cell line-specific variance in screen quality is also observed across DepMap using the Avana library (Supplementary Fig. 4c)^17^.

As the MAPK signaling pathway is composed of paralog redundancies at multiple nodes, we next explored the synergistic dependencies observed in IPC298 harboring *NRAS*^*Q61L*^ mutation. As expected, we identified members of the pathway including RAF (BRAF-RAF1), MEK (MAP2K1-MAP2K2), ERK (MAPK1-MAP3), and RSK2 (RPS6KA1, RPSKA3, and RPS6KB1) family exhibiting synergistic dependencies (Fig. 5b, c). In addition, we also observed paralogs that negatively regulate the pathway, including the 14-3-3 genes (YWHAE-YWHAZ), MEK phosphatases (PPP2CA-PPP2CB), and ERK phosphatases (DUSP4-DUSP6), displaying synergistic dependencies through hyperactivation of the pathway (Fig. 5c)^10,24,25^. These data suggest that both pan-lethal gene pairs, as well as selective essential gene are robustly found in these screens. It is notable that BRAF/RAF1 are essential in the *NRAS*-mutant setting as highly selective dual BRAF/RAF1 inhibitors have enter clinical trials in both the *BRAF*-mutant resistant setting and in the setting of *NRAS*-mutant tumors^26^.

## Discussion

In this study, we assessed the robustness of ten pooled dual-knockout systems and found that combinations of alternative spCas9 tracrRNAs (VCR1-WCR3) are associated with superior performance in both the single-gene and combinatorial dropout screens. The improvements can be attributed to several aspects including sgRNA design and the more divergent tracrRNA sequences between the two sgRNAs.

While single-gene CRISPR libraries can tolerate some level of poor-performing sgRNAs, as long as a majority exhibit consistency for individual genes, combinatorial libraries are less tolerant of poor-performing guides as individual sgRNAs are utilized multiple times. For example, in our library design, if three out of twelve sgRNAs between the two genes were ineffective, we observe a bifurcation of the dataset amongst the dual knockouts. To this end, although various sgRNA prediction tools are improving, utilization of “pre-validated” sgRNAs selected from hundreds of pooled genome-scale CRISPR-Cas9 screens resulted in better performance^27^. We expect additional data from gene tiling screens would additionally improve future sgRNA designs^22,28^.

Cas12a possesses the potential for higher order of combinatorial screens and have been used in prior studies utilizing the canonical Cas12a PAM sequence (TTTV)^13,15^. In this study, we generated the enCas12a library based on recent reports of broader range of PAM sites^12,14^. This expansion is potentially important in that it could allow a much broader range of guides to be designed compared to the restrictive TTTV sequence. Based on our screening results, we recapitulate prior studies that canonical TTTV PAM sequence is significantly more efficient than non-canonical PAM sequences (Supplementary Fig. 3a). Nonetheless, cells infected with sgRNAs targeting pan-essential genes using the VCR1-WCR3 tracrRNA combination (Supplementary Fig. 3c) exhibited stronger guide depletion compared to the optimal TTTV PAM by enCas12a (Supplementary Fig. 3a). Cas12a performance will most likely progress as sgRNA design principles improve and the availability of large-scale Cas12a screens become available.

Although tracrRNA modifications like tetraloop extensions have shown improved on-target activity^29,30^, the high sequence variability throughout the two tracrRNAs was observed to be main factor in improving the variability, sgRNA dropout effect, and balance between the two genes. For example, in cases where we utilized the WCR3 tracrRNA using the same crRNA sequence and promoter, we observed a drastic difference in dropout effect when paired with WCR2 or VCR1. Sequencing through the tracrRNA suggested that similar sequences between the WCR2 and WCR3 tracrRNAs led to higher levels of recombination rate in both the pDNA and gDNA. In conclusion, we combined improved sgRNA design and Cas9 tracrRNA combinations which outperformed other systems based on multivariate parameters. Further analyses comparing other recent combinatorial technologies like Cas Hybrid for Multiplexed Editing and screen Application (CHyMErA; Cas9-Cas12 hybrid) or CRISPR interference platforms are needed to understand how these systems perform against the VCR1-WCR3 tracrRNA combination^11,31^. Nonetheless, the system described here for dual-gene knockout screens represents a robust and powerful methodology to examine genetic interactions between multiple gene pairs or to reduce genome-scale pooled libraries^15,32^.

## Methods

### Cell lines

The MELJUSO (DSMZ; #ACC74), PK1 (RIKEN; #RCB1972), and IPC298 (DSMZ; #ACC251) cell lines were collected by the CCLE^33^. All cell lines were maintained in RPMI (Gibco) medium supplemented with 10% fetal bovine serum (FBS), penicillin (100 µg ml^−1^), streptomycin (100 µg ml^−1^), and l-glutamine (292 µg ml^−1^; Gibco).

### Cas9 and Cas12 library production

Cas9 oligonucleotide pools were synthesized (Twist Bioscience) with BsmBI sites and appropriate overhang sequences spanning the two 20-nt crRNAs. Two BbsI sites were designed between the two crRNAs. The final oligonucleotide sequence was thus: 5′-AGGCACTTGCTCGTACGACGCGTCTCGCACCG [crRNA, 20nt] GTTTCAGTCTTCCGGCGAAGACACCTGAAAC [reverse complement crRNA, 20nt] CGGGAAGAGACGTTAAGGTGCCGGGCCCACAT-3′. Primers were used to amplify the oligonucleotide pools using 25 μL 2x NEBnext PCR master mix (New England Biolabs), 2 μL of oligonucleotide pool (~40 ng), 5 μL of primer mix at a final concentration of 0.5 μM, and 18 μL water. PCR cycling conditions: 30 s at 98 °C, 30 s at 53 °C, 30 s at 72 °C, for 24 cycles. For cloning the pooled crRNA sequences, dsDNA was purified by spin-column (Qiagen), digested with Esp3I (Fisher Scientific), and ligated into the Esp3I-digested pWRS1001 vector using 100 cycles of Golden Gate assembly with 150 ng insert and 500 ng vector using Esp3I and T7 ligase. pWRS1001 is a lentiviral vector with U6 and H1 promoters expressing the two sgRNAs and a short EF1a promoter (EFS) expressing puromycin resistance cassette adapted from pPapi (also known as pXPR_207; Addgene 96921) by introducing silent mutations at all BbsI sites and removing saCas9. For the incorporation of the tracrRNAs, the purified BbsI digested tracrRNA fragment was cloned in between the dual crRNAs by the second round of Golden Gate cloning. The ligation product was isopropanol precipitated and electroporated into Stbl4 electrocompetent cells (Life Technologies) and grown at 30 °C for 16 h on agar with 100 μg mL^−1^ carbenicillin. Colonies were scraped and plasmid DNA (pDNA) was prepared (HiSpeed Plasmid Maxi, Qiagen). Cas12a oligonucleotides were synthesized (Twist Biosciences) with the following template: 5′-AGGCACTTGCTCGTACGACGCGTCTCAAGAT [enCas12a crRNA, 23 nt] TAATTTCTACTGTCGTAGAT [enCas12a crRNA, 23nt] TTTTTTGAATcGAGACGTTAAGGTGCCGGGCCCACAT-3′. The 134nt oligos included an engineered variant of the wild-type direct repeat for AsCas12a between the two 23-nt-long crRNAs^12^. The oligonucleotides pool was amplified and cloned into pRDA_052 (Addgene #136474) as described above. The guide expression vector pRDA_052 was digested with BsmBI (New England Biolabs), de-phosphorylated with Antarctic phosphatase (New England Biolabs), and concentrated using PCR cleanup columns (Life Technologies).

### Virus production

For pooled library lentiviral production, 18 × 10^6^ HEK293T cells were seeded per 175-cm^2^ tissue culture flask for 16 h. Transfection was performed using TransIT-LT1 (Mirus) transfection reagent according to the manufacturer’s protocol. Briefly, one solution of 6 mL Opti-MEM and 305uL of LT1 was combined with DNA mixture of the packaging plasmid pCMV_VSVG (Addgene 8454, 5ug), psPAX2 (Addgene 12260, 50 μg), and the transfer vector (40ug). The solutions were incubated at room temperature for 30 min, added dropwise to the surface of the HEK293T cells, and transferred to a 37 °C incubator for 8 h, after which the media was removed and replaced with DMEM + 30% FBS media. The virus was harvested and filtered 36 h after the last media change.

### Paralog library screening

Cas9 stable cell lines were transduced with a blasticidin-resistant pLX_311-Cas9 vector (Addgene 96924). enCas12a stable cell lines were transduced with a blasticidin-resistant pRDA_174 (Addgene 136476). Prior to the screening, Cas9/enCas12a-expressing cell lines were selected with blasticidin then transduced with the CRISPR library viruses in three biological replicates to achieve a representation of 750 cells per sgRNA and at low multiplicity of infection (MOI; 0.3–0.5). Cell lines were transduced in 12-well plates at 3.0 × 10^6^ cells per well in the presence of polybrene with an appropriate volume of virus. The plates were centrifuged at 931 × g for 2 h then transferred to a 37 °C incubator for 18 h. Cells were split and treated with puromycin for 3 days. Throughout the screen, cells were split and replated to maintain representation. Cell counts were taken at each passage to monitor growth. Cells were pelleted by centrifugation, resuspended in PBS, and processed for genomic DNA isolation using the NucleoSpin Blood L (TakaraBio) as per the manufacturer’s instructions.

### sgRNA PCR for Illumina sequencing

PCR of gDNA and pDNA was performed in multiple 100 μL reactions (total volume) containing a maximum of 10 μg gDNA or 1 ng pDNA. DNA was PCR-amplified and barcoded with P5/P7 primers (Integrated DNA Technologies) using Titanium Taq polymerase (Takara) according to the manufacturer’s instructions. Briefly, per one reaction, a PCR master mix consisted of 2 μL 50× Titanium Taq polymerase, 10 μL of 10× Titanium Taq reaction buffer, 8 μL of dNTP, 0.5 μL of P5 stagger primer mix (stock at 100 μM concentration), and 19.5 μL water. Each well consisted of 50 μL gDNA or pDNA plus water, 40 μL of PCR master mix, and 10 μL of a uniquely barcoded P7 primer (stock at 5 μM concentration). PCR cycling conditions for Cas9 libraries were: an initial 5 min at 95 °C; followed by 30 s at 95 °C, 30 s at 55 °C, 20 s at 72 °C, for 22 cycles; and a final 10-min extension at 72 °C. PCR cycling conditions for Cas12 libraries were: an initial 5 min at 95 °C; followed by 30 s at 95 °C, 30 s at 52.5 °C, 30 s at 72 °C, for 28 cycles; and a final 10-min extension at 72 °C. Samples were purified with Agencourt AMPure XP SPRI beads according to the manufacturer’s instructions (Beckman Coulter, A63880). Samples were sequenced on a NextSeq 75 bp × 2 paired-end or 150 bp × 2 paired-end for Cas9 screens and NextSeq 150 bp × single-end for Cas12a screens (Illumina).

### Cas activity assay

Cas9 and Cas12a activities were measured using an EGFP fluorescent reporter assay. Briefly, vector constitutively expressing destabilized EGFP and sgRNA targeting EGFP were used to assess Cas9 (pXPR_047; Addgene #107145) or Cas12a (pRDA_221; Addgene #169142) activities. Cas9 or enCas12a engineered cell lines were transduced with pXPR_047 or pRDA_221 lentivirus and passaged for five days in appropriate concentration of puromycin. Cas9 or Cas12a activities were measured by the percentage of EGFP-negative cells using FACS (CytoFLEX; Beckman-Coulter Life Sciences).

### Selection of genes and paralog pairs

Corresponding paralog pairs for curated sets of genes were obtained from ENSEMBL (release 93). For single-gene positive controls, pan-essential genes which were selected from Hart essential gene list that demonstrated strong dependency in both RNAi (probability >0.5 in >50% cell lines with DEMETER2^34^) and CRISPR (probability >0.8 in >90% cell lines in Achilles Avana Database)^35^. Similarly, nonessential genes were picked from Hart nonessential list that demonstrate low dependency profiles in RNAi (probability <0.2 in >95% cell lines with DEMETER2 estimations) and CRISPR (probability <0.2 in >96% cell lines in Achilles Avana Database). The dual-knockout positive controls were selected based on the CCLE and DepMap data as previously described^10^. Briefly, a binary loss-of-function matrix (cell lines by genes) was constructed by logic combination of low copy number or deleterious mutation using the CCLE and DepMap data. Low copy number is defined by the relative copy number (log_2_) of at least 2 standard deviation for each cell line below the mean of all cell lines for a given gene. Deleterious mutations were predicted by frameshift indel or nonsense single-nucleotide variants in whole-exome or whole-genome sequencing. Differential dependency was tested using the DepMap CRISPR dependency scores (probability of dependency) for 659 cell lines and 13,301 genes, which were included in the CRISPR dataset, loss-of-function feature matrix, and had at least one annotated paralog. For each gene target in the CRISPR dataset, we performed a two-class comparison of the dependency scores, grouped according to the gene paralog pairs (binary loss-of-function feature). Significant differences in dependency were assessed by a one-sided Kolmogorov–Smirnov test of the continuous dependency score and a one-sided Fisher’s exact test of the dependency score binarized at 0.5. This resulted in two *P* values for each dependency paralog pair, which were adjusted using the Benjamini–Hochberg method. Selected synthetic lethal paralog pairs had an FDR  < 0.05 for at least one of the two significance tests and a mean percentile of both tests in the top 10%. The negative control consists of paralog pairs both with low mRNA expression based on CCLE profiling (log2(TPM + 1) <0.1 in 90% of CCLE cell lines).

### Selection of sgRNAs for Cas9 and enCas12a

Cas9 and enCas12a sgRNA selections were curated from the top 100 sgRNAs from the ranked databases (maximize on-target and minimize off-target) in Genetic Perturbation Platform (GPP)^12,20^. For Cas9, additional sgRNAs exhibiting consistent performance with other sgRNAs targeting the same gene across cell lines with Achilles Avana datasets were supplemented. The guide consistency metric was determined by training the random forest. The agreement metrics for 5000 pairs of guides targeting the same gene was first generated, and then 5000 pairs targeting two random genes. The random forest was trained to use these metrics to discriminate the first group from the second. With the agreement score available for each pair of sgRNAs within a gene, we identified the group of highly consistent (agreement score > 0.7) sgRNAs with at least 3 members and completed the initial guide selection pool for Cas9 by combining this with GPP databases. Off-targets were further removed from the initial list with Cas-OFFinder by searching genome-wide off-targets with 0 and 1 mismatch. We set stringent off-target filtering criteria by eliminating guides with 0 mismatch (PAM NRG for Cas9, all three tiers of PAM sites for enCas12a) and 1 mismatch (PAM NGG for Cas9, all 3 tiers of PAM sites for enCas12a). For genes without sufficient sgRNAs (*N* = 6 per gene) after this filtering step, we loosened the criteria with tolerating 1 mismatch and less efficient PAM site for enCas12a. For Cas9 sgRNAs, we further incorporated our findings from the previous paralog library in terms of guide efficacy. Guides produced gene phenotypes inconsistent with the estimated gene effect from Achilles Avana were further removed from the selection pool. Lastly, we ranked our sgRNA pool by prioritizing sgRNAs validated with empirical screen evidence (consistent performance in Achilles Avana for Cas9), sgRNAs targeting PFAM domains while keeping a high rank from GPP databases (within top 20 suggested pick order), and the rest of sgRNAs available from GPP list based on provided pick orders. PFAM domains were annotated by using the genetic coordinate intersection function with bedtools intersect^36^. The list of Cas9 and Cas12a sgRNA sequences, source, PFAM annotation, and pairs are listed in Supplementary Data 5–8.

### Calculation of LFC, synergy, and FDR

The GEMINI R package was used to calculate raw counts (Supplementary Data 9), LFCs counts (Supplementary Data 10) and sensitive synergy scores counts (Supplementary Data 11) with their corresponding FDRs^37^. We refer to the mean of LFCs of guide pairs targeting the same gene pair as the raw LFC, the mean of LFCs targeting a gene paired with negative control (AAVS1) would be treated as the single-gene effect while double gene effects were computed as mean of LFCs targeting a paralog pair. GEMINI computes the sensitive synergy score by comparing the double gene effect to the more lethal single-gene effect produced by genes in the pair. For calculation of the FDR, GEMINI uses a set of non-synergistic pairs in each cell line, as described in the previous section, and constructs the null distributions by fitting a Gaussian mixture model to synergy scores associated with the gene pairs. *P* value for each gene pair is calculated as the right-tail probability that the null distribution generates a synergy score greater than the score of that pair and FDRs are subsequently calculated based on Benjamini–Hochberg procedure^38^.

### Separation of control sets

Null-normalized median difference (NNMD) and the Area Under the ROC Curve (AUC-ROC) were used to assess the separation of two control populations. Both measures are derived from the sensitive synthetic lethality score calculated as defined in GEMINI. The independence of two control groups is assumed when implementing NNMD. In terms of generating the ROC curve, we constructed the confusion matrix, calculated True Positive Rate (TPR) and False Positive Rate (FPR) at various thresholds of the sensitive synthetic lethality scores. The selection of control populations at single-gene and gene pair knockout space were as described in previous method sections.

### tracrRNA design and selection

WCR2 and WCR3 were obtained from multiplexed Perturb-seq system ensuring at most 20 bases of continuous sequence homology to each other (originally referred to as cr2 and cr3, respectively)^19^. SCR27 and SCR43 were obtained from generation of nonrepetitive extra-long sgRNA arrays (ELSA) examined in *Escherichia coli* (originally referred to as handle sgRNA #27 and #43, respectively)^18^. To enable cloning of SCR27 and SCR43 into the pWRS1001 vector, part of the stem-loop was reverted back to the endogenous SpCas9 tracrRNA sequence which differs from the published sequence^18^. VCR1 is a tracrRNA sequence of high sequence diversity previously shown to maintain high cutting efficiencies^21^. The diversity was achieved by extending both the stem-loop and hairloop #1.

### Statistic and reproducibility

All CRISPR screens were performed in three biological replicates. PCR analysis for Supplementary Fig. 1j was performed in two biological replicate and a representative image is shown. Cas9 and Cas12a activity assays for Supplementary Fig. 4b were performed in three biological replicates. Pearson correlation was used for Fig. 2e, Fig. 4d, and Supplementary Fig. 2a, b. The Wilcoxon test was used for Supplementary Fig. 3c. Statistical and bioinformatic data analyses were performed using R (V4.0.3) and Rstudio (V1.2.5042) using the following R packages: pbapply(v1.5-0), pbmcapply(v1.5.0), RColorBrewer(v1.1–2), eulerr(v6.1.1), ggtext(v0.1.1), ggpubr(v0.4.0), ggplot2(v3.3.5), openxlsx(v4.2.4), readr(v2.0.2), purrr(v0.3.4), tidyr(v1.1.4), dplyr(v1.0.7), magrittr(v2.0.1), mgsub(v1.7.3), glue(v1.5.0), Biostrings(v2.58.0), XVector(v0.30.0), IRanges(v2.24.1), S4Vectors(v0.28.1), BiocGenerics(v0.36.1), stringr(v1.4.0).

### Reporting summary

Further information on research design is available in the Nature Research Reporting Summary linked to this article.

## Supplementary information

Supplementary Information

Reporting Summary

Description of Additional Supplementary Files

Supplementary Data 1

Supplementary Data 2

Supplementary Data 3

Supplementary Data 4

Supplementary Data 5

Supplementary Data 6

Supplementary Data 7

Supplementary Data 8

Supplementary Data 9

Supplementary Data 10

Supplementary Data 11

## Data Availability

The FASTQ files generated in this study have been deposited in the Sequence Read Archive (SRA) database under accession code PRJNA792754. The raw data for running the analysis pipeline are available on Figshare at https://figshare.com/articles/dataset/Zipped_Raw_data/19565902. The processed data and relevant controls in this study are provided in the Supplementary Information and Source Data file. The additional dataset used in analyses include: paralog identification (ENSEMBL; release 93), PFAM Identification (PFAM EMBL-EBI; version 33.1), sgRNA off-target (Cas-OFFinder); Cancer Cell Line Encyclopedia (DepMap; public 21q4). Source data are provided with this paper.

## Source data

Source Data

## Code availability

All computational analyses were performed in R (version 3.5.2). Source codes are available at https://github.com/sellerslab/CombiMiniLib and 10.5281/zenodo.6436145.
