# Supplementary material for: Comparative optimization of combinatorial CRISPR screens
Source: Nat Commun. 2022 May 5;13:2469. doi: 10.1038/s41467-022-30196-9 (PMC9072436; doi:10.1038/s41467-022-30196-9)
Supplement: Supplementary file 1 — Supplementary Information [file 41467_2022_30196_MOESM1_ESM.pdf]

Supplementary Information

Comparative Optimization of Combinatorial CRISPR Screens

Ruitong Li, Olaf Klingbeil, Davide Monducci, Michael J. Young, Diego J. Rodriguez, Zaid Bayyat, Joshua M. Dempster, Devishi Kesar, Xiaoping Yang, Mahdi Zamanighomi, Christopher R. Vakoc, Takahiro Ito, William R. Sellers

a

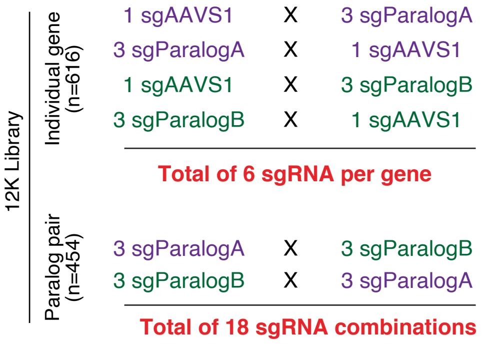

b

tracrRNA	Source
SPCR1	Original *S. pyogenes*
VCR1 (left)	Modified tracrRNA variant
VCR1 (right)	Modified tracrRNA variant
WCR2	cr2 - Adamson et. al *Cell* (2017)
WCR3	cr3 - Adamson et. al *Cell* (2017)
SCR27	cr27 - Adapted from Reis et. al *Nature Biotechnology* (2019)
SCR43	cr43 - Adapted from Reis et. al *Nature Biotechnology* (2019)

c

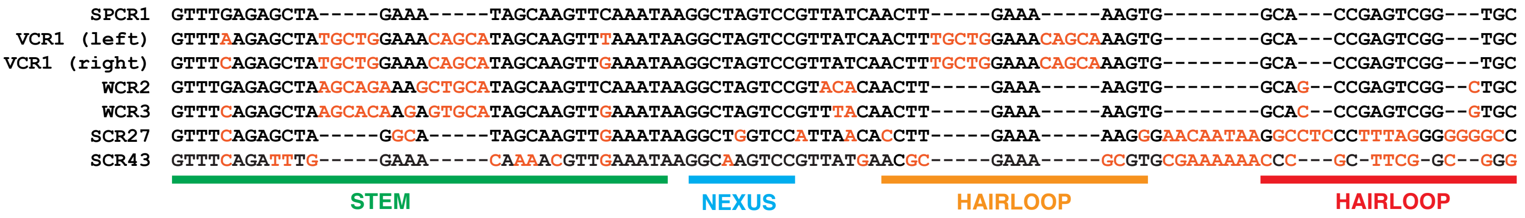

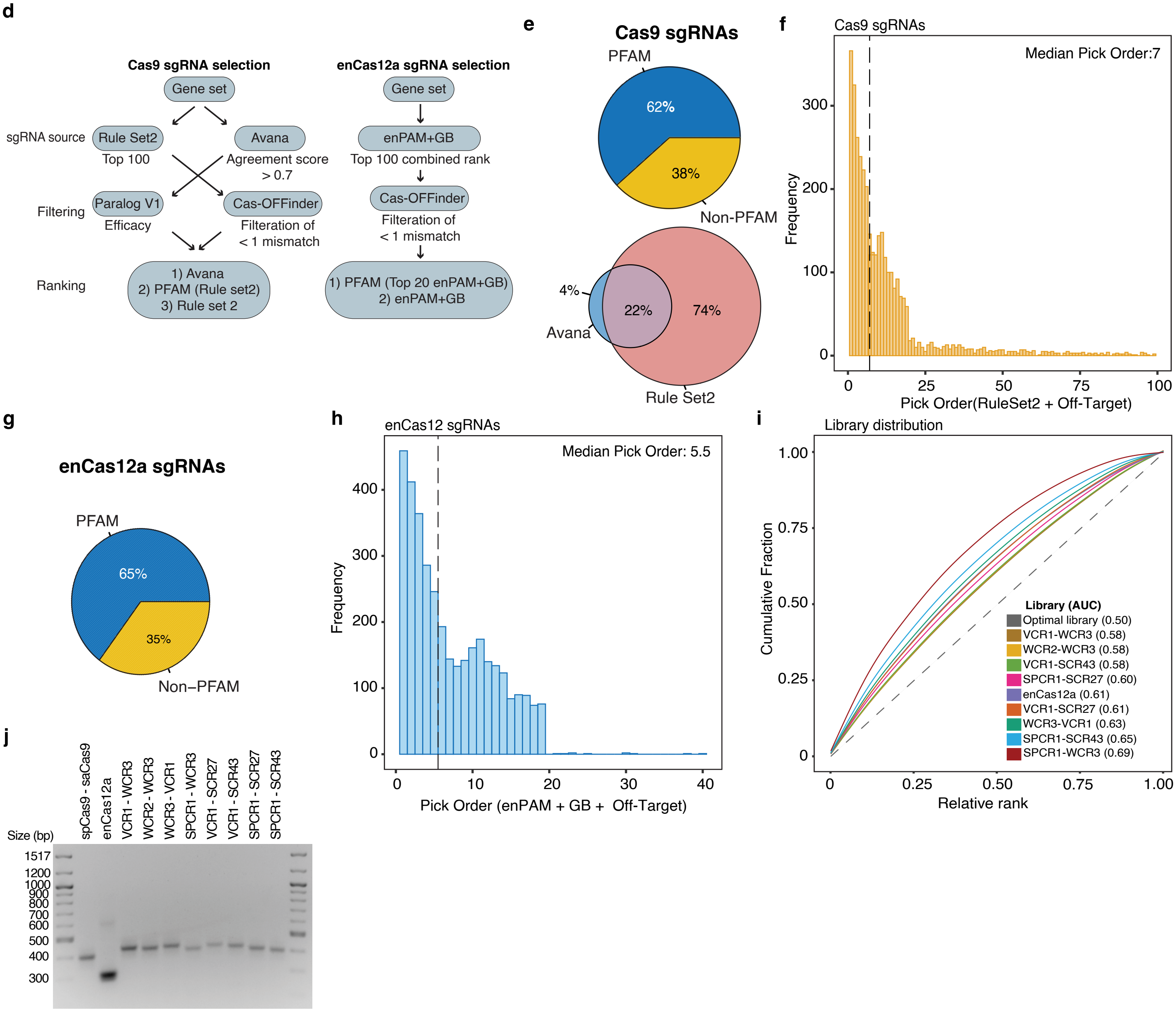

Supplementary Fig. 1: Composition of the combinatorial libraries. (**a**) Schematic of the library design. The nine 12K libraries generated targeting 616 individual genes with 6 sgRNAs coupled with AAVS1 sgRNA cutting control (3 sgRNA in right and left position). Dual knockouts of 454 paralog pairs by 18 sgRNA combinations are utilized. (**b**) Source and nomenclature of alternative tracrRNA sequences. (**c**) Sequence of alternative tracrRNAs. (**d**) Source and prioritization of sgRNAs for Cas9 and enCas12a. (**e**) Pie chart depicting the percentage of Cas9 sgRNAs targeting PFAM and non-PFAM domain (*top*). Venn diagram of sgRNAs from Avana library, Rule Set 2 or both (*bottom*). (**f**) Distribution of Cas9 sgRNAs based on Rule Set 2 Pick Order. Dotted line represents the median Pick Order. (**g**) Pie chart depicting the percentage of enCas12a sgRNAs targeting PFAM and non-PFAM domain. (**h**) Distribution of enCas12a sgRNAs based on Pick Order. Dotted line represents the median Pick Order. (**i**) Area-under-the-curve (AUC) analysis for library distribution from 12K library pDNAs. (**j**) PCR analysis of the pDNA using PCR primers and conditions used to amplify libraries for sequencing analysis. Source data are provided in the Source Data File.

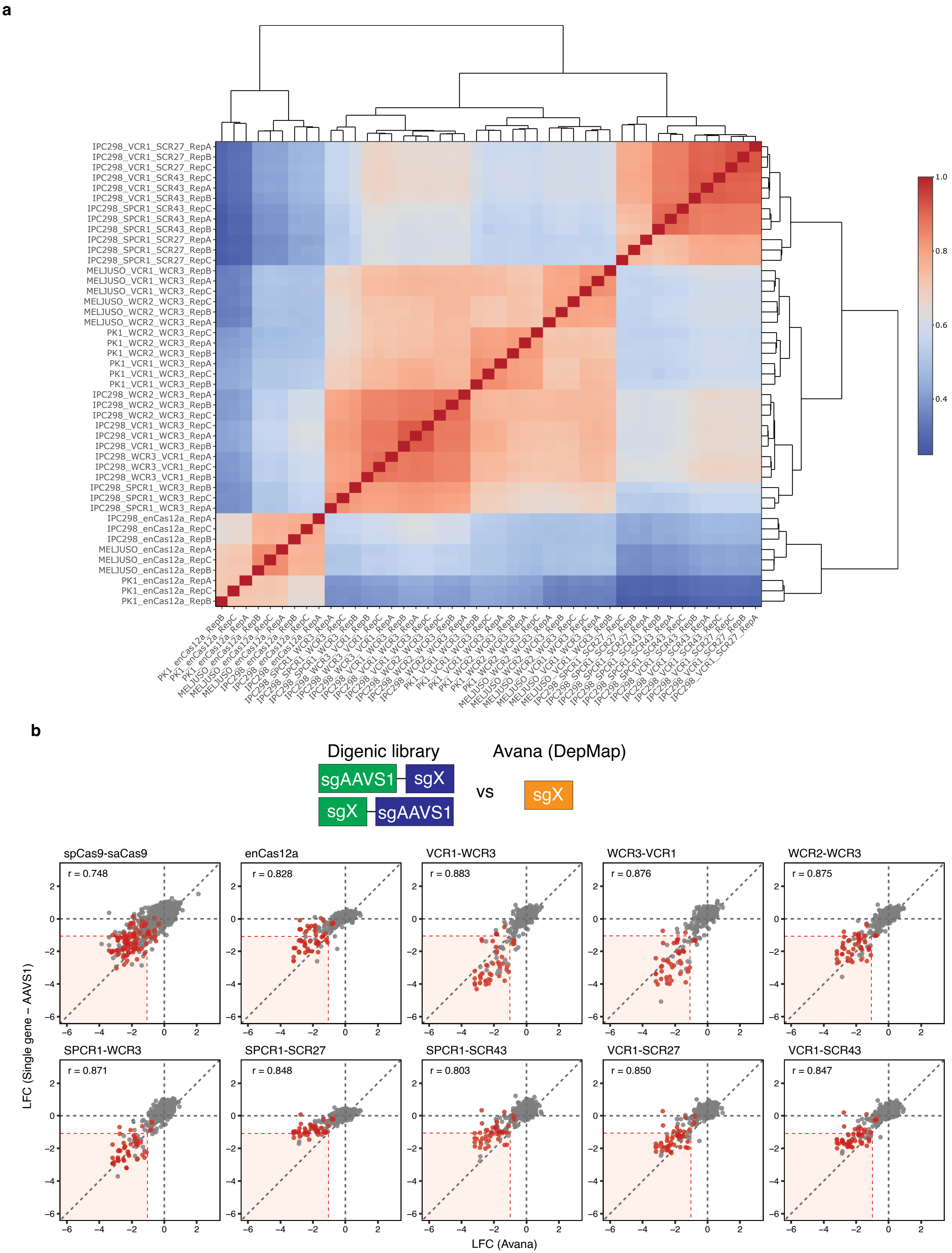
a
IPC298_VCR1_SCR27_RepA
IPC298_VCR1_SCR27_RepB
IPC298_VCR1_SCR27_RepC
IPC298_VCR1_SCR43_RepC
IPC298_VCR1_SCR43_RepA
IPC298_VCR1_SCR43_RepB
IPC298_SPCR1_SCR43_RepC
IPC298_SPCR1_SCR43_RepA
IPC298_SPCR1_SCR43_RepB
IPC298_SPCR1_SCR27_RepA
IPC298_SPCR1_SCR27_RepB
IPC298_SPCR1_SCR27_RepC
MELJUSO_VCR1_WCR3_RepB
MELJUSO_VCR1_WCR3_RepA
MELJUSO_VCR1_WCR3_RepC
MELJUSO_WCR2_WCR3_RepC
MELJUSO_WCR2_WCR3_RepB
MELJUSO_WCR2_WCR3_RepA
PK1_WCR2_WCR3_RepC
PK1_WCR2_WCR3_RepA
PK1_WCR2_WCR3_RepB
PK1_VCR1_WCR3_RepA
PK1_VCR1_WCR3_RepC
PK1_VCR1_WCR3_RepB
IPC298_WCR2_WCR3_RepA
IPC298_WCR2_WCR3_RepB
IPC298_WCR2_WCR3_RepC
IPC298_VCR1_WCR3_RepC
IPC298_VCR1_WCR3_RepA
IPC298_VCR1_WCR3_RepB
IPC298_WCR3_VCR1_RepA
IPC298_WCR3_VCR1_RepC
IPC298_WCR3_VCR1_RepB
IPC298_SPCR1_WCR3_RepB
IPC298_SPCR1_WCR3_RepC
IPC298_SPCR1_WCR3_RepA
IPC298_enCas12a_RepA
IPC298_enCas12a_RepC
IPC298_enCas12a_RepB
MELJUSO_enCas12a_RepA
MELJUSO_enCas12a_RepC
MELJUSO_enCas12a_RepB
PK1_enCas12a_RepA
PK1_enCas12a_RepC
PK1_enCas12a_RepB
1.0
0.8
0.6
0.4
b
Digenic library
sgAAVS1 sgX
sgX sgAAVS1
vs
Avana (DepMap)
sgX
spCas9-saCas9
r = 0.748
enCas12a
r = 0.828
VCR1-WCR3
r = 0.883
WCR3-VCR1
r = 0.876
WCR2-WCR3
r = 0.875
SPCR1-WCR3
r = 0.871
SPCR1-SCR27
r = 0.848
SPCR1-SCR43
r = 0.803
VCR1-SCR27
r = 0.850
VCR1-SCR43
r = 0.847
LFC (Single gene – AAVS1)
LFC (Avana)
2
0
−2
−4
−6
−6
−4
−2
0
2

Supplementary Fig. 2: Quality controls for combinatorial systems and the screen performance. (**a**) Pearson correlation heatmap of LFCs between all pairwise combinations of the 12K library screens across three cell lines (IPC298, MELJUSO, PK1). (**b**) Scatterplot of the LFC for each gene against the LFC from Avana DepMap CRISPR dataset in IPC298. Pan-essential genes are annotated in red. Source data are provided in the Source Data File.

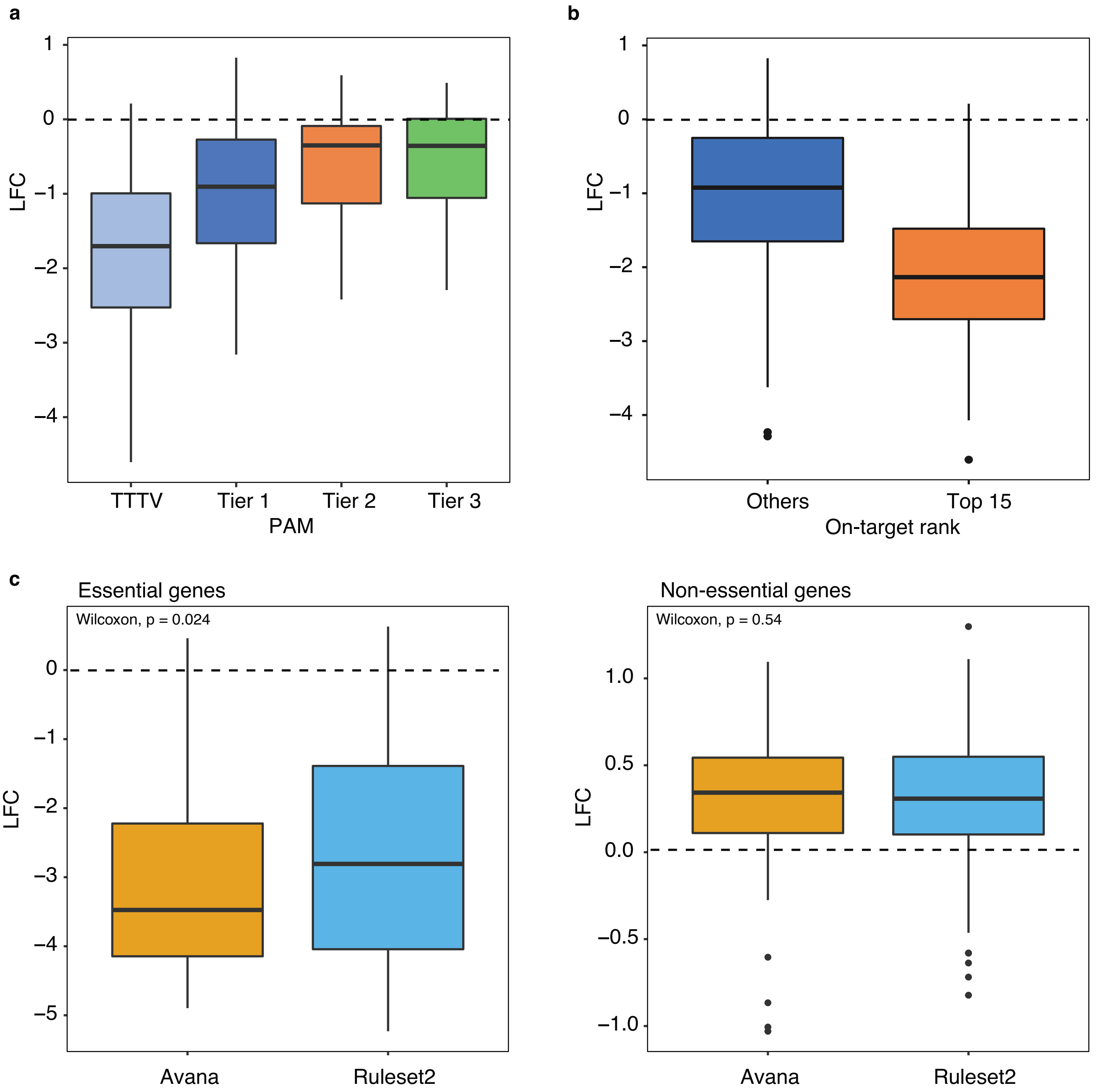
a
b
c
LFC
1
0
−1
−2
−3
−4
TTTV
Tier 1
Tier 2
Tier 3
PAM
Others
Top 15
On-target rank
Essential genes
Wilcoxon, p = 0.024
−5
Avana
Ruleset2
Non-essential genes
Wilcoxon, p = 0.54
1.0
0.5
0.0
−0.5
−1.0

Supplementary Fig. 3: Evaluation of enCas12a and Cas9 sgRNA design. (**a**) Activity of enCas12a sgRNA targeting essential genes binned by PAM tiers. (**b**) Same as (a) binned by on-target rank. (**c**) Activity of Cas9 sgRNA targeting essential (*left*) or non-essential (*right*) genes grouped by sgRNAs source (DepMap Avana screens or Rule Set 2). Significant difference is determined using Wilcoxon test. For the boxplots, the centerline, lower hinge, and upper hinge correspond to the 50th, 25th, and 75th percentiles, respectively. The upper and lower whiskers extend from the upper and lower hinges to the largest and smallest values no further than 1.5 * IQR (interquartile range). Source data are provided in the Source Data File.

a

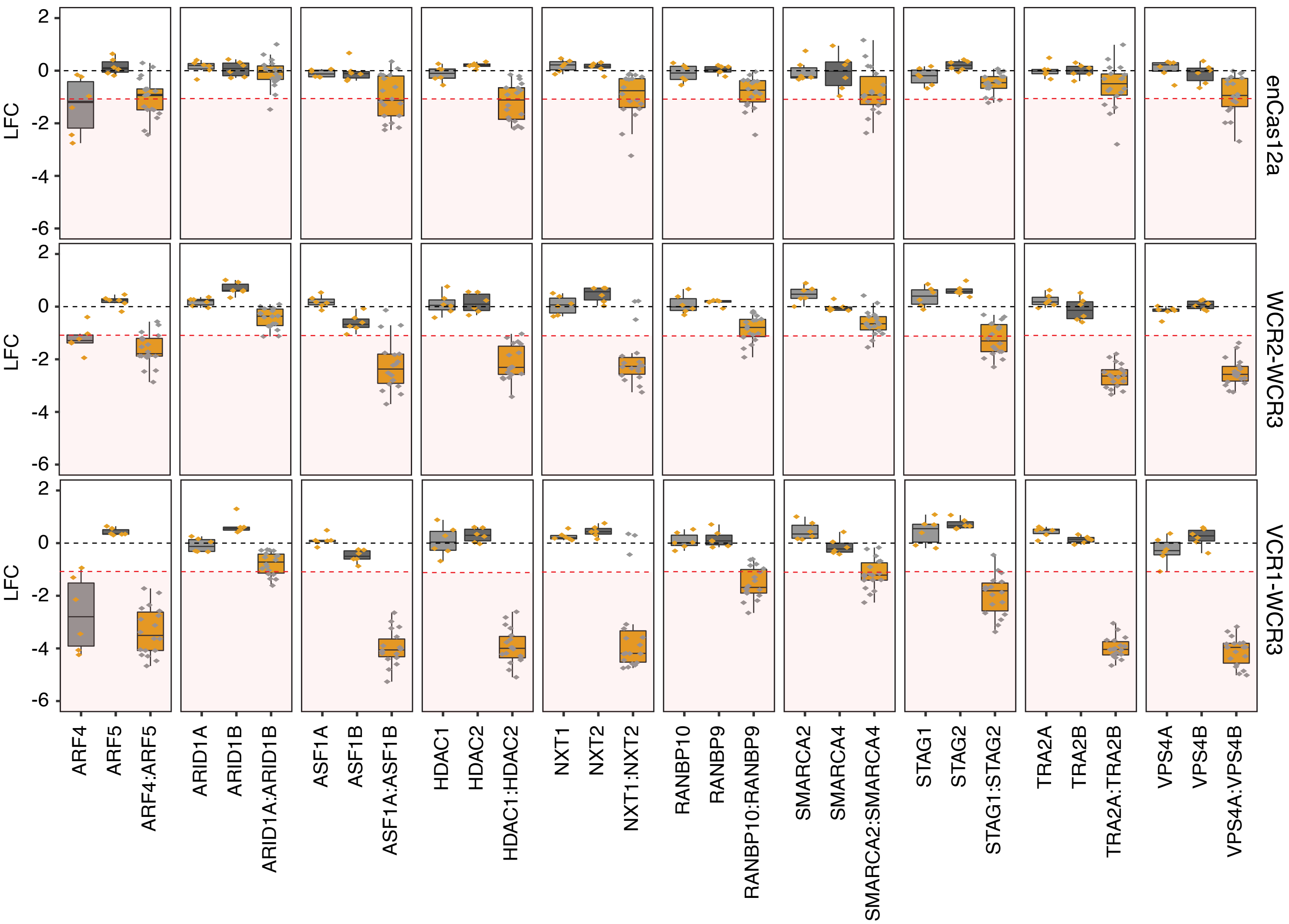
LFC
enCas12a
WCR2-WCR3
VCR1-WCR3
2
0
-2
-4
-6
ARF4
ARF5
ARF4:ARF5
ARID1A
ARID1B
ARID1A:ARID1B
ASF1A
ASF1B
ASF1A:ASF1B
HDAC1
HDAC2
HDAC1:HDAC2
NXT1
NXT2
NXT1:NXT2
RANBP10
RANBP9
RANBP10:RANBP9
SMARCA2
SMARCA4
SMARCA2:SMARCA4
STAG1
STAG2
STAG1:STAG2
TRA2A
TRA2B
TRA2A:TRA2B
VPS4A
VPS4B
VPS4A:VPS4B

b

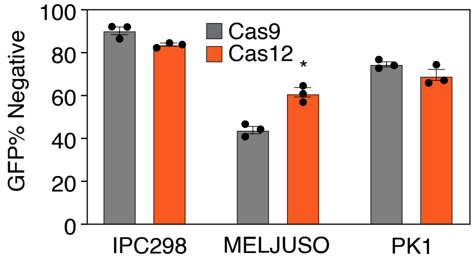
GFP% Negative
100
80
60
40
20
0
Cas9
Cas12
*
IPC298
MELJUSO
PK1

c

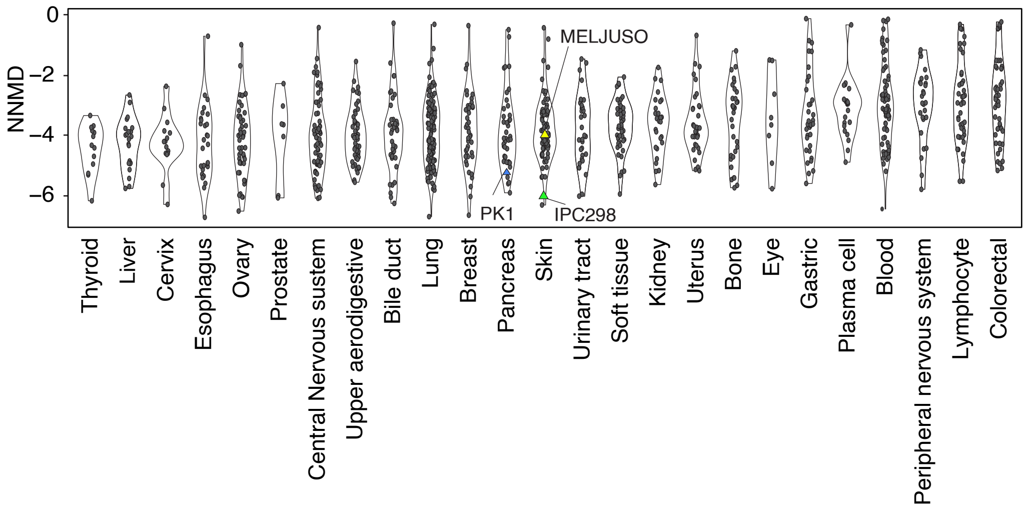
NNMD
0
-2
-4
-6
MELJUSO
PK1
IPC298
Thyroid
Liver
Cervix
Esophagus
Ovary
Prostate
Central Nervous sustem
Upper aerodigestive
Bile duct
Lung
Breast
Pancreas
Skin
Urinary tract
Soft tissue
Kidney
Uterus
Bone
Eye
Gastric
Plasma cell
Blood
Peripheral nervous system
Lymphocyte
Colorectal

Supplementary Fig. 4: Comparison of LFC of pan-essential paralog pairs. (**a**) LFC of individual sgRNA combinations for single-gene and dual knockout of pan-essential paralog pairs in IPC298. The centerline, lower hinge, and upper hinge correspond to the 50th, 25th, and 75th percentiles, respectively. The upper and lower whiskers extend from the upper and lower hinges to the largest and smallest values no further than 1.5 * IQR (interquartile range). n=3 biological replicates; 6 independent sgRNAs per gene and 18 independent sgRNA combination per gene pairs. (b) Cas9 and Cas12 activity assay assessed by percentage of GFP negative cells measured by FACS in Cas9 or enCas12a-stably expressing cells transduced with vector ectopically expressing EGFP and sgEGFP. The data are shown as mean ± SD (n=3 biologically independent replicates). *P-value≤ 0.001, two-way ANOVA. (c) Separation between non- and pan-essential genes computed by NNMD score across CCLE. Source data are provided in the Source Data File.
