# Supplementary material for: Comparative optimization of combinatorial CRISPR screens
Source: Nat Commun. 2022 May 5;13:2469. doi: 10.1038/s41467-022-30196-9 (PMC9072436; doi:10.1038/s41467-022-30196-9)
Supplement: Supplementary file 2 — Reporting Summary [file 41467_2022_30196_MOESM2_ESM.pdf]

nature portfolio

Corresponding author(s): William R Sellers, Takahiro Ito

Last updated by author(s): Apr 11, 2022

Reporting Summary

Nature Portfolio wishes to improve the reproducibility of the work that we publish. This form provides structure for consistency and transparency in reporting. For further information on Nature Portfolio policies, see our Editorial Policies and the Editorial Policy Checklist.

Statistics

For all statistical analyses, confirm that the following items are present in the figure legend, table legend, main text, or Methods section.

n/a	Confirmed	
☐	☒	The exact sample size (n) for each experimental group/condition, given as a discrete number and unit of measurement
☐	☒	A statement on whether measurements were taken from distinct samples or whether the same sample was measured repeatedly
☐	☒	The statistical test(s) used AND whether they are one- or two-sided<br>*Only common tests should be described solely by name; describe more complex techniques in the Methods section.*
☒	☐	A description of all covariates tested
☐	☒	A description of any assumptions or corrections, such as tests of normality and adjustment for multiple comparisons
☐	☒	A full description of the statistical parameters including central tendency (e.g. means) or other basic estimates (e.g. regression coefficient) AND variation (e.g. standard deviation) or associated estimates of uncertainty (e.g. confidence intervals)
☐	☒	For null hypothesis testing, the test statistic (e.g. F, t, r) with confidence intervals, effect sizes, degrees of freedom and P value noted<br>*Give P values as exact values whenever suitable.*
☒	☐	For Bayesian analysis, information on the choice of priors and Markov chain Monte Carlo settings
☒	☐	For hierarchical and complex designs, identification of the appropriate level for tests and full reporting of outcomes
☐	☒	Estimates of effect sizes (e.g. Cohen's d, Pearson's r), indicating how they were calculated

Our web collection on statistics for biologists contains articles on many of the points above.

Software and code

Policy information about availability of computer code

Data collection	All sequencing were performed using NextSeq 500 (Illumina).
Data analysis	Software used: R (V4.0.3), Rstudio (V1.2.5042).<br><br>R Packages: pbapply(v1.5-0), pbmcapply(v1.5.0), RColorBrewer(v1.1-2), eulerr(v6.1.1), ggtext(v0.1.1), ggpubr(v0.4.0), ggplot2(v3.3.5), openxlsx(v4.2.4), readr(v2.0.2), purrr(v0.3.4), tidyr(v1.1.4), dplyr(v1.0.7), magrittr(v2.0.1), mgsub(v1.7.3), glue(v1.5.0), Biostrings(v2.58.0), XVector(v0.30.0), IRanges(v2.24.1), S4Vectors(v0.28.1), BiocGenerics(v0.36.1), stringr(v1.4.0).<br><br>All computational analyses were performed in R (version 3.5.2). Source codes are available at https://github.com/sellerslab/CombiMiniLib and https://doi.org/10.5281/zenodo.6436145.

For manuscripts utilizing custom algorithms or software that are central to the research but not yet described in published literature, software must be made available to editors and reviewers. We strongly encourage code deposition in a community repository (e.g. GitHub). See the Nature Portfolio guidelines for submitting code & software for further information.

Data

Policy information about availability of data

All manuscripts must include a data availability statement. This statement should provide the following information, where applicable:

- Accession codes, unique identifiers, or web links for publicly available datasets
- A description of any restrictions on data availability
- For clinical datasets or third party data, please ensure that the statement adheres to our policy

- FASTQ files generated in this study have been deposited in the Sequence Read Archive (SRA) database under accession code PRJNA792754 [https://www.ncbi.nlm.nih.gov/bioproject/PRJNA792754]. The raw data for running the analysis pipeline are available on figshare at https://figshare.com/articles/dataset/Zipped_Raw_data/19565902. The processed data and relevant controls in this study are provided in the Supplementary Information/Source Data file.
- Database for paralog identification: ENSEMBL (release 93)
- Database for PFAM identification: Pfam EMBL-EBI (version 33.1)
- Cas OFFinder: http://www.rgenome.net/cas-offinder/
- Processed LFC and GEMINI scores: Provided in supplementary data
- All genomic data from Cancer Cell Line Encyclopedia (CCLE) available at (https://portals.broadinstitute.org/ccle/data). DepMap 21q4 was used for all analyses.

Field-specific reporting

Please select the one below that is the best fit for your research. If you are not sure, read the appropriate sections before making your selection.

[x] Life sciences [ ] Behavioural & social sciences [ ] Ecological, evolutionary & environmental sciences

For a reference copy of the document with all sections, see nature.com/documents/nr-reporting-summary-flat.pdf

Life sciences study design

All studies must disclose on these points even when the disclosure is negative.

Sample size	All CRISPR screens were all performed in biological triplicates. This is the standard in the field and the high correlation between individual cell lines (Extended Data Fig. 2a) suggests adequate sample size was used. Prior studies comparing large-scale CRISPR screens across different CRISPR libraries, sites, duration and NGS methodologies using biological duplicates or triplicates showed high agreement across hundreds of cell lines (https://www.nature.com/articles/s41467-019-13805-y)
Data exclusions	sgRNA pair targeting AAVS1 (sgAAVS1-sgAAVS1) was removed from the analyses due to unexpected high enrichment as a result of uncoupling effects (describe in Hedge et. al, Plos One 2018).
Replication	The correlation of CRISPR screen performance between biological triplicate across different libraries and cell lines are shown in Extended Data Fig. 2a.). PCR analysis for Supplementary Fig. 1j was performed in biological duplicate and Cas9/Cas12a activity assays for Supplementary Fig. 4b were performed in biological triplicates and show high reproducibility.
Randomization	No clinical research study with group randomization and blinding is involved in this study. Same screen conditions and sequencing preparation protocol have been used for all Cas9 and enCas12a screens. Sequencing depth has been controlled by normalizing the counts by total in each biological replicate.
Blinding	Samples were not blinded. Knowledge of the reagents and cell lines were necessary to perform the experiments given different culture condition and transduction efficiencies.

Reporting for specific materials, systems and methods

We require information from authors about some types of materials, experimental systems and methods used in many studies. Here, indicate whether each material, system or method listed is relevant to your study. If you are not sure if a list item applies to your research, read the appropriate section before selecting a response.

Materials & experimental systems

n/a	Involved in the study	
x		Antibodies
	x	Eukaryotic cell lines
x		Palaeontology and archaeology
x		Animals and other organisms
x		Human research participants
x		Clinical data
x		Dual use research of concern

Methods

n/a	Involved in the study	
x		ChIP-seq
x		Flow cytometry
x		MRI-based neuroimaging

Eukaryotic cell lines

Policy information about cell lines

Cell line source(s)	The MELJUSO (DSMZ), PK1 (RIKEN), and IPC298 (DSMZ) cell lines were collected by the Cancer Cell Line Encyclopedia (Broad-Novartis) and all Cas9 engineered by expression of pLX311-Cas9 (addgene #1181018) by the Genetic Perturbation Platform (Broad Institute). 293T cell line was obtained from ATCC. The detailed information of the aforementioned cell lines can also be found at depmap.org.
Authentication	All cell lines were fingerprinted by SNP arrays and STR profiling.
Mycoplasma contamination	Cell lines were tested negative for mycoplasma.
Commonly misidentified lines (See ICLAC register)	Project Achilles performed fingerprinting to ensure the identify of the screened cell lines. All misidentified cell lines were removed from the final dataset. See McDonald, E.R., et al., Cell. (2017) for details regarding the cell lines in Project DRIVE. Our experiments included no commonly misidentified cell lines as per ICLAC version 9.
