# Supplementary material for: Comparative optimization of combinatorial CRISPR screens
Source: Nat Commun. 2022 May 5;13:2469. doi: 10.1038/s41467-022-30196-9 (PMC9072436; doi:10.1038/s41467-022-30196-9)
Supplement: Supplementary file 3 — Description of Additional Supplementary Files [file 41467_2022_30196_MOESM3_ESM.pdf]

Title: Supplementary Data 1:
Description: List of pan-essential genes

Title: Supplementary Data 2:
Description: List of non-essential genes

Title: Supplementary Data 3:
Description: List of pan-essential pairs

Title: Supplementary Data 4:
Description: List of non-essential pairs

Title: Supplementary Data 5:
Description: Cas9 sgRNA sequences and source

Title: Supplementary Data 6:
Description: Cas9 sgRNA pair combinations

Title: Supplementary Data 7:
Description: Cas12a sgRNA and PFAM annotation

Title: Supplementary Data 8:
Description: Cas12a sgRNA pair combinations

Title: Supplementary Data 9:
Description: Raw counts from CRISPR screens

Title: Supplementary Data 10:
Description: LFC from CRISPR screens

Title: Supplementary Data 11:
Description: GEMINI sensitive synergy score from CRISPR screens
